# Longitudinal Associations Between Community Violence Exposure and Mental Health Problems in Inner-City Youth: Ethnicity and Gender Perspectives

**DOI:** 10.1177/08862605231158754

**Published:** 2023-03-13

**Authors:** Vladislav Ruchkin, Johan Isaksson, Andrew Stickley, Mary Schwab-Stone

**Affiliations:** 1 Uppsala University, Sweden; 2 Yale University School of Medicine, New Haven, CT, USA; 3 Sala Forensic Psychiatric Clinic, Sweden; 4 National Institute of Mental Health, National Center of Neurology and Psychiatry, Kodaira, Tokyo, Japan; 5 Södertörn University, Huddinge, Sweden

**Keywords:** community violence exposure, mental health problems, longitudinal, inner-city, ethnicity, gender, adolescents

## Abstract

There is a lack of agreement on whether children and adolescents with different cultural/ethnic backgrounds react to trauma in a similar fashion. This study adds to the existing literature by providing ethnicity and gender perspectives on the longitudinal associations between the degree of community violence exposure (CVE) and mental health problems in U.S. inner-city youth. The study was conducted on a representative sample of predominantly ethnic minority youth (*N* = 2,794; 54.1% female; age 11–16 years old (*M* [*SD*] = 12.77 [1.29]); 60.0% African-American, 26.1% Hispanic American, 13.9% White). Self-reported information was obtained on CVE in year 1 and on mental health problems (depressive symptoms, posttraumatic stress, alcohol use, and conduct problems) in year 1 and year 2. Multivariate analyses of covariance (MANCOVA) were used to compare mental health problems in youth from the three ethnic groups in relation to the different degrees of CVE experienced one year prior, while controlling for their baseline mental health problem levels, age, and socio-economic status. Mental health problems in year 2 increased in a similar fashion in relation to the degree of severity of CVE in year 1 in all three ethnic groups. The interaction effects suggested a gender-specific response to CVE, where girls in the three ethnic groups reported higher levels of depression and posttraumatic stress in relation to the same degree of CVE, as compared to boys. Adolescents from different ethnic backgrounds respond similarly to differing degrees of CVE with an increase in mental health problems over time. In response to a similar degree of exposure, girls tend to experience greater levels of internalizing problems than boys. Timely recognition of traumatic exposure and associated mental health problems is important for early prevention and intervention strategies.

## Introduction

Mortality data of U.S. children and adolescents from 2016 showed that firearm-related injuries were the second leading cause of death, responsible for 15% of deaths (Cunningham et al., 2018), while more recent data suggest that almost 2 million youths between the ages of 10 and 14 were admitted to emergency units in 2020 as a result of violence-related injuries (Centers for Disease Control and Prevention, 2020). Indeed, community violence exposure (CVE) represents one of the most common adverse childhood experiences (Carlson et al., 2020) and is defined as exposure to a violence-related act within one’s home, school, or neighborhood between individuals who are unrelated, and who may or may not know each other (Krug et al., 2002).

Research on CVE has differentiated between its two types or degrees of severity/physical proximity, namely the witnessing of violence and violent victimization (Fowler et al., 2009; Kennedy & Ceballo, 2014; Schwab-Stone et al., 1999), where witnessing generally involves viewing acts of violence or threats of it, whereas victimization refers to being a target of an intentionally harmful act (e.g., Fowler et al., 2009). It has been estimated that CVE may affect as many as two out of every three youth in the United States (Finkelhor et al., 2013). Adolescents and young adults between the ages of 12 to 24 are more likely than individuals in other age groups to experience CVE (Finkelhor et al., 2009), and older youth tend to be exposed to violence particularly often (Klaus & Rennison, 2002). Research suggests that CVE is conditioned by complex interactions between several demographic characteristics, including gender, age, ethnicity, and urban/rural status (Hammond & Arias, 2011; Weist et al., 2001).

Youth from low-income, ethnic minority urban communities are at increased risk for CVE (e.g., Fowler et al., 2009). Disproportionate exposure to such neighborhood risk factors as pervasive, enduring poverty (Sampson et al., 1997), concentrated disadvantage, and a shortage of youth services (Zimmerman & Messner, 2013), all significantly increase the probability of CVE. Studies estimate that 50% to 96% of ethnic minority male adolescents may witness violence, while up to one-third may be directly victimized in their communities (Fehon et al., 2001; Fowler et al., 2009; Gorman-Smith et al., 2004), although some research suggests even higher rates of violent victimization (Gaylord-Harden et al., 2011). Even when controlling for neighborhood disadvantage and household income (Crouch et al., 2000; Zimmerman & Messner, 2013), as well as for demographic characteristics, such as age and gender (Weist et al., 2001), ethnic minority youth still experience disproportionally higher levels of CVE. Research suggests that they are twice as likely to witness a shooting or a stabbing as White youth even within the same school district (Schwab-Stone et al., 1995), and the odds of Hispanic-American and African-American youth experiencing CVE are 74% and 112% higher respectively than those of their White counterparts (Zimmerman & Messner, 2013). It has been thus suggested that ethnic minority youth residing in urban, low-income communities are more at risk for CVE than any other population in the United States (Aisenberg & Herrenkohl, 2008).

A better understanding of the effects of CVE in ethnically diverse populations is particularly important, given their disproportionally high rates of exposure and potential differences in associated mental health problems (e.g., Fowler et al., 2009; James et al., 2018). In addition, while the prevalence of posttraumatic stress may be consistent across different populations exposed to similar traumatic events, what is considered a traumatic event in one setting may be differently perceived in another (Herbert & Forman, 2010). Although research has explored factors related to CVE in ethnically diverse populations, and suggested that both the prevalence of CVE and its mental health outcomes may vary by culture/ethnicity (e.g., Schwab-Stone et al., 2013; Rosenthal & Wilson, 2006; Vermeiren et al., 2003), as yet, only a few studies have directly compared the effects of CVE in different ethnic groups (e.g., Chen, 2010; Chen et al., 2020; López et al., 2017), and the findings have been rather inconclusive, meaning that it still remains unclear whether racial/ethnic differences exist with regards to how CVE correlates with adolescent mental health outcomes (Chen et al., 2020). This may be an important oversight, considering the general lack of agreement on whether individuals with a different cultural/ethnic background respond to trauma in a similar way (Schwab-Stone et al., 2001).

Both the rates of CVE and its psychological consequences tend to vary by gender. Studies have shown that males are more likely to encounter violent events than females (Finkelhor et al., 2015; Koposov et al., 2021; Weist et al., 2001). Specifically, research has demonstrated that the odds of being exposed to violence are 51% higher for boys than for girls (Zimmerman & Messner, 2013), and that male gender is the single most significant predictor of being a victim of a shooting among urban high school students (Chandler et al., 2011). Similarly, within urban ethnic minority youth, male adolescents are much more likely to experience CVE than female adolescents (Elsaesser & Voisin, 2015; Lambert et al., 2012). At the same time, while males are more often exposed to violence, females who experience trauma report more distress and impairment compared to traumatized males (Giaconia et al., 1995), are more often diagnosed with posttraumatic stress disorder (PTSD) (Alisic et al., 2014; Garza & Jovanovic, 2017), and are more likely to experience internalizing mental health problems (Miliauskas et al., 2022). Despite this, to the best of our knowledge, no previous study has explored the role of gender differences in mental health outcomes in response to community violence by ethnicity.

Notably, the levels of psychosocial problems in youths exposed to CVE seem to correlate with the proximity to violence or degree of exposure (Buka et al., 2001; Fowler et al., 2009; Stansfeld et al., 2017), where a greater degree of CVE (from witnessing to victimization) is associated with higher levels of posttraumatic stress, depression, and antisocial behavior (e.g., Goldner et al., 2015; Schwab-Stone et al., 2013). Other studies (e.g., Shields et al., 2010) have similarly indicated that victimization is associated with a greater number of psychological problems than witnessing, and a dose-dependent effect of CVE on depressive symptoms, posttraumatic stress, and anxiety has been reported (Gaylord-Harden et al., 2011; Goldner et al., 2015).

Hence, there is a need to further investigate the longitudinal effects of differing degrees of traumatic exposure in urban adolescent populations (Lambert et al., 2010), as such appraisals may provide a better insight into the dynamics of trauma in relation to different ethnic/cultural backgrounds. Similarly, given the gender-specific patterns of trauma reactions, a lack of studies on the gender-related dynamics of CVE in ethnically diverse populations appears to be a significant omission.

The aims of this study were therefore to: (a) determine the ethnicity- and gender-specific prevalence of CVE in a representative sample of inner-city ethnic minority youth, (b) examine whether adolescents with different ethnic backgrounds who experienced the same degree of CVE at baseline would report a similar increase in the severity of mental health problems one year later (controlling for their baseline levels), and whether similar effects were observed for increasing levels of CVE, and (c) investigate whether these effects vary by gender, both in the overall sample and within different ethnic groups.

Based on previous research we hypothesized that at each level of CVE girls would report higher rates of internalizing (depression, posttraumatic stress) symptoms than boys, and that the longitudinal associations between the degree of CVE and mental health problems would follow a similar gender-specific pattern in all ethnic groups.

## Methods

### Participants

The survey was conducted with all eligible students in the New Haven (CT) public school system, including students in alternative programs and bilingual classes (17 public middle and high schools). In the spring of year one, 3,639 students in the age group 11 to 16 years completed the survey. This sample was then followed until the next survey administration one year later. Seventy-eight percent of the original sample (*n* = 2,847) completed the survey in year 2. High attrition in longitudinal studies of urban ethnic minority adolescents is common (e.g., Patel et al., 2003), and related to the high mobility level of families and high rates of school drop-out. Results indicated that the 792 students who dropped out (compared to the 2,847 remaining students) were older (*M* [*SD*] = 13.47 [1.56] vs. 12.83 [2.08], *t* = 7.97, *p* < .001), more likely to be male (413 [52.1%] vs. 1,369 [48.1%], χ^2^ = 4.09, *p* **<** .05), Hispanic-American (248 [32.0%] vs. 708 [25.3%]) and less likely to be African-American (422 [54.5%] vs. 1,717 [61.5%], χ^2^ = 15.04, *p* **<** .001). These drop-out students did not differ from the follow-up group in their CVE rates, but reported higher levels of depression (*M* [*SD*] = 5.34 [4.70] vs. 4.74 [4.29], *t* = 3.32, *d* = 0.133, *p* **<** .01), posttraumatic stress (*M* [*SD*] = 23.51 [14.20] vs. 22.04 [13.25], *t* = 2.66, *d* = 0.107, *p* **<** .05), conduct problems (*M* [*SD*] = 3.72 [4.79] vs. 2.80 [4.09], *t* = 5.24, *d* = 0.207, *p* **<** .001), and alcohol use (*M* [*SD*] = 1.27 [2.21] vs. 0.77 [1.68], *t* = 6.64, *d* = 0.255, *p* **<** .001). These findings suggest that attrition was selective across nearly all key variables.

Give our intent to examine ethnicity as a main variable in the analyses, the sample was restricted to participants with an African-American, Hispanic-American, and White ethnic background, which resulted in the exclusion of 53 (1.9%) subjects from other ethnic groups (i.e., Asian-American and Other).

Finally, 265 (9.6%) adolescents had missing data. In relation to this, multiple imputation was undertaken (i.e., the data were imputed 25 times) using the Markov Chain Monte Carlo method with a Predictive mean matching model for continuous variables. No categorical background variables lacked data, as information on these variables was obtained from the school registry.

The final sample (*N* = 2,794); 54.1% female; age at baseline 11–16 years (*M* [*SD*] = 12.77 [1.29]) was predominantly comprised of minority ethnicities (60.0% African-American, 26.1% Hispanic American, 13.9% White), an accurate reflection of the local public-school population (New Haven Public Schools, 2019). A majority of the students came from single parent families (54.3%). The sample population was predominantly socio-economically disadvantaged, as reflected by the large proportion (over 71%), who qualified for free/reduced lunch status at either point of the data collection. Over 80% of students’ caregivers had the equivalent of a high school education or beyond.

### Procedure

Parents were informed of the survey at the time of school registration, received a letter about the survey 2 weeks prior to its administration, and were offered the opportunity to decline participation. The passive informed consent procedure was approved by the university’s institutional review board and considered as an appropriate ethical procedure by the state legislature. Prior to the survey’s administration, students were read a detailed assent form outlining their participation with the assurance of confidentiality, and were asked to sign it to indicate assent (parent and child refusals were less than 1%). Students completed the survey in their classrooms during a single class period on a regular school day. Trained administrators read all the questions aloud, while students followed along with their copies of the survey, reading questions to themselves and circling responses in the booklet. A second administrator was available, providing help to individual students on request. Surveys were administered in English and Spanish. All students in the respective grades attending schools on the day of the survey’s administration were eligible to participate. Make-up administrations were performed at each school within one month of the initial administration for those who had been absent.

### Measures

*CVE* was assessed in year 1 with items derived from the Screening Survey of Exposure to Community Violence (Richters & Martinez, 1993). The students were asked “about things that may happen to people in some neighborhoods”. Using a 5-point response format (ranging from None [0] to 10 or more times [4]) the students described whether in the past year they had witnessed and/or been victimized by six types of violence (been beaten up or mugged, threatened with serious physical harm, shot or shot at with a gun, attacked or stabbed with a knife, chased by gangs or individuals, or seriously wounded in an incident of violence). Three groups were formed according to the reported degree of violence exposure. Those who did not report any witnessing and victimization episodes were considered as the *non-exposed group* (categorized with a ‘0’). Those, who reported at least one episode of witnessing, but no episodes of victimization were regarded as the *witnessing group* (1). Finally, those, who reported at least one episode of victimization, regardless of witnessing, were considered as the *victimization group* (2).

*Depressive symptoms* were assessed in years 1 and 2 using an adaptation of the *Center for Epidemiologic Studies-Depression Scale* (Radloff, 1977), which has demonstrated excellent psychometric properties with adolescent populations (e.g., Carpenter et al., 1998). The scale consists of 10 statements (e.g., “I felt like crying”; “I felt that many bad things were my fault”), inquiring about the presence of depressive symptoms during the past month, which are assessed using a three-point scale (“*Not true*” [0]; “*Somewhat true*” [1]; or “*Certainly true*” [2]). The total score ranges from 0 to 20, with higher scores indicating more depressive symptoms. McDonald’s omega was .82 for African-American, .84 for White and .81 for Hispanic-American students.

*Posttraumatic stress* was assessed in years 1 and 2 with the Child Post-Traumatic Stress-Reaction Index (Pynoos et al., 1987), a 20-item index assessing the frequency of posttraumatic stress symptoms on a 5-point scale, ranging from “*Never*” (0) to “*Most of the time*” (4). The total score could range from 0 to 80, with higher scores corresponding closely with a clinical diagnosis of PTSD (Steinberg et al., 2004). McDonald’s omega was .92 for African-American, .89 for White and .84 for Hispanic-American students.

*Alcohol use* was assessed in years 1 and 2 with three items derived from the Monitoring the Future Scale (Johnston et al., 1990). The instrument included three items assessing the use of three alcoholic beverages (beer, wine, and hard liquor) during the past 30 days on a four-point scale, ranging from “0 times” (0) to “6 or more times” (3). The possible scale score ranges from 0 to 9, with higher scores indicating greater alcohol use. McDonald’s omega was .87 for African-American, .86 for White and .82 for Hispanic-American students.

*Conduct Problems* were evaluated with six items in years 1 and 2 (Schwab-Stone et al., 1999) describing relatively mild behavior problems, such as lying to a teacher or a parent, staying out all night without permission, shoplifting, damaging property, and skipping school. The respondents were asked to report on a 5-point scale how many times (ranging from “0 times” [0] to “5 or more times” [4]) they were involved in these behaviors during the past year. The scale total sum score can range from 0 to 24, with higher scores indicating more conduct problems. McDonald’s omega was .88 for African-American, .89 for White and .86 for Hispanic-American students.

*Proxy for Socio-economic Status (SES).* Eligibility for free (2) or reduced lunch (1) in year 1 was used as an index of SES and could hence vary between 0 and 2 with higher scores indicating worse SES. Students were eligible if their family’s income was less than 185% of the federal poverty threshold.

### Statistical Analyses

The Statistical Package for the Social Sciences (SPSS-26.0) was used to analyze the data. Chi-square (with Cramer’s V) and independent sample *t*-tests (with Cohen’s *d* effect sizes) were performed when making univariate comparisons of demographic characteristics. General linear models multivariate analysis of covariance (MANCOVA) was used to determine main and interaction effects across the fixed factors of CVE (0, 1, 2) (as described earlier), gender (girls = 0, boys = 1), and ethnicity (African-Americans [1], White [2], and Hispanic-Americans [3]), while adjusting for SES, age, and mental health problems in year 1. MANCOVA analyses were chosen over univariate analyses of covariance (ANCOVAs) because evidence suggests that mental health problems commonly co-occur and thus, there is a need to evaluate the individual weight of each particular problem, while adjusting for potential confounders. Further, in our analyses we were interested in assessing the between-subject differences (MANCOVA), as opposed to within-subjects differences (which can be assessed by repeated measures MANCOVA) in the dependent variables. In other words, we were not that interested in seeing how the score for each dependent variable changes over time in relation to the degree of exposure to violence, race, and gender, but rather, in evaluating how the race and gender groups differed from each other in relation to all of the dependent variables, depending on their degree of CVE and after controlling for the initial levels of the dependent variables.

MANCOVA analyses were conducted with mental health problems (depression, posttraumatic stress, alcohol use, and conduct problems) as dependent variables reported one year after the assessment of CVE. Thus, we used a 3 (CVE) × 2 (Gender) × 3 (Ethnicity) design for assessing differences in mental health problems in year 2, while controlling for the baseline levels of the same problems in year 1. The unique contribution of each of the three fixed factors, the six covariates (SES proxy, age, depression, posttraumatic stress, alcohol use, and conduct problems), and the four interaction terms were evaluated through follow-up between-subject tests and unstandardized parameter estimates derived from the MANCOVA. Results are presented as means (*M*) and standard deviations (*SD*), and for individual outcomes as Wilks’ lambda, *F*(*df*), *p* and partial eta squared (η^2^) that reflects the unique amount of variance explained by each predictor variable. Cohen (1988) provided points of reference to define small (η^2^ = .01), medium (η^2^ = .06), and large (η^2^ = .14) effects.

## Results

### Gender and Ethnic Differences in the Prevalence of CVE

The prevalence (%) of CVE by gender within each ethnic group is presented in Table 1. Witnessing and victimization rates differed by gender, with higher levels of victimization and lower levels of witnessing in boys, as compared to girls, in all ethnic groups. As regards ethnicity, White students reported a lower prevalence of witnessing than the other ethnic groups, but the prevalence of victimization did not differ between the groups.

**Table 1.** Prevalence of the Different Degrees of CVE by Ethnicity and Gender (%).

		Ethnicity			Comparisons by Ethnicity
Degree of CVE:		African-American (*n* = 17,17)	White (*n* = 369)	Hispanic-American (*n* = 708)	(Chi-square, Cramer’s V, *p*)
Witnessing	Boys	39.5^a^	23.7^b^	34.6^a^	16.18; .110; <.001
	Girls	52.4^a^	33.3^b^	50.5^a^	23.35; .127; <.001
Comparisons by gender (Chi-square, Cramer’s V, *p*)		28.79; .129; <.001	4.15; .106; .042	18.37; .161; <.001	
	Total	46.3^a^	28.7^b^	42.8^a^	38.29; .117; <.001
Victimization	Boys	47.4^a^	46.9^a^	41.0^a^	4.09; .055; .130
	Girls	26.2^a^	28.1^a^	24.7^a^	.766; .023; .682
Comparisons by gender (Chi-square, Cramer’s V, *p*)		83.28; .220; <.001	13.90; .194; <.001	21.28; .173; <.001	
	Total	36.2^a^	37.1^a^	32.6^a^	3.37; .035; .185

*Note*. The same superscript letter denotes categories whose column proportions do not differ significantly from each other at the .05 level.

### Longitudinal Associations Between CVE and Mental Health Problems

When evaluating the differences in mental health problems by CVE (see Table 2 and Supplemental figures for descriptive statistics (*M* [*SD*]) by gender and ethnicity, and Table 3 for the main and interaction effects, and for the tests of between-subjects effects), the main effect for the model was significant (Wilks’ lambda = .964; *F* (4, 2,767) = 25.99; *p* < .001; η^2^ = .036). With regard to specific effects, the main effect for CVE was significant, with the levels of all mental health problems increasing in line with increasing degrees of CVE. The main effect for Gender was also significant, indicating higher levels of internalizing mental health problems in girls and higher levels of externalizing problems in boys (see Tables 2 and 3). The main effect for Ethnicity was significant, with follow-up tests demonstrating differences in depressive symptoms and alcohol use by ethnic group. The main effect for age was also significant and was related to higher levels of depressive symptoms and alcohol use with increasing age. The main effect for the SES proxy (free lunch) was not significant, suggesting there were no differences in mental health problems by SES.

**Table 2.** Mental Health Problems (*M* [*SD*]) in Year 2 by Ethnicity and CVE in Year 1 in Boys (B) and Girls (G).

Ethnicity		African-American			White			Hispanic-American		
CVE		No (*n* = 300)	W (*n* = 795)	V (*n* = 622)	No (*n* = 126)	W (*n* = 106)	V (*n* = 137)	No (*n* = 174)	W (*n* = 303)	V (*n* = 231)
Depressive	B	2.86 (4.01)	3.00 (3.48)	3.78 (3.75)	4.19 (4.99)	3.08 (3.58)	3.84 (3.78)	1.87 (2.44)	2.91 (2.97)	4.72 (4.58)
Symptoms	G	3.99 (3.90)	4.88 (4.38)	6.60 (4.73)	4.72 (4.46)	6.49 (4.88)	8.79 (5.98)	5.40 (5.08)	5.47 (4.23)	7.54 (4.58)
Posttraumatic	B	14.87 (11.22)	17.67 (11.13)	20.63 (12.00)	15.04 (12.65)	15.44 (12.51)	17.59 (12.19)	10.64 (6.35)	16.45 (11.58)	22.42 (14.57)
Stress	G	17.88 (11.45)	20.96 (12.05)	27.80 (14.17)	18.54 (13.25)	24.14 (17.18)	31.80 (16.68)	20.00 (13.53)	22.77 (12.24)	30.44 (13.18)
Alcohol	B	.53 (1.33)	.55 (1.27)	1.01 (2.00)	1.12 (2.52)	1.56 (2.41)	2.16 (2.89)	.37 (1.18)	1.02 (2.04)	1.72 (2.57)
Use	G	.62 (1.47)	.73 (1.49)	1.15 (1.75)	.74 (1.51)	.97 (1.76)	1.69 (2.31)	.48 (1.16)	1.18 (1.88)	1.59 (2.37)
Conduct	B	2.72 (4.20)	4.45 (5.16)	6.53 (6.39)	4.69 (6.34)	6.81 (6.39)	7.68 (6.89)	1.73 (2.73)	4.20 (5.67)	6.64 (7.10)
Problems	G	3.26 (4.37)	3.93 (4.12)	5.53 (5.27)	3.30 (4.56)	4.11 (4.54)	5.93 (5.96)	2.64 (3.59)	4.62 (5.50)	5.32 (4.80)

*Note*. CVE = Community violence exposure; No = No CVE; W = Witnessing; V = Victimization.

**Table 3.** Main and Interaction Effects and Effect Sizes for Each Dependent Variable (Mental Health Problems) (η^2^, *p*).

	Main and Interaction Effects (Wilks’ lambda; *F*[*df*]; η^2^; *p*)	Depressive symptoms	Posttraumatic stress	Alcohol use	Conduct problems
Age	.968; 23.08 (4, 2,767); .032; *p* < .001	.014, <.001	.000, ns	.010, <.001	.000, ns
Free lunch	.999; 1.02 (4, 2,767); .001; ns	.000, ns	.001, ns	.000, ns	.000, ns
Depressive symptoms	.923; 57.88 (4, 2,767); .077; *p* < .001	.072, <.001	.020, <.001	.000, ns	.001, ns
Posttraumatic stress	.886; 89.03 (4, 2,767); .114; *p* < .001	.016, <.001	.107, <.001	.000, ns	.001, ns
Alcohol use	.888; 87.08 (4, 2,767); .112; *p* < .001	.000, ns	.009, ns	.104, <.001	.003, <.01
Conduct problems	.858; 114.05 (4, 2,767); .142; *p* < .001	.002, <.05	.001, ns	.013, <.001	.137, <.001
Gender	.964; 25.79 (4, 2,767); .036; *p* < .001	.024, <.001	.015, <.001	.002, <.05	.004, <.001
CVE	.990; 3.64 (8, 5,534), .005; *p* < .001	.001, ns	.003, <.01	.002, <.05	.006, <.001
Ethnicity	985; 5.25 (8, 5,534); .008; *p* < .001	.006, <.001	.001, ns	.008, <.001	.002, ns
CVE × Gender	.994; 2.23 (8, 5,534); .003; *p* < .05	.004, <.01	.002, <.05	.000, ns	.001, ns
CVE × Ethnicity	.993; 1.24 (16, 8,453); .002; ns	.001, ns	.001, ns	.002, ns	.002, ns
Gender × Ethnicity	991; 3.26 (8, 5,534); .005; *p* < .001	.003, <.05	.004, <.01	.002, <.05	.003, <.05
CVE × Gender × Ethnicity	.993; 1.28 (16, 8,453); .002; ns	.006, <.01	.004, <.01	.000, ns	.001, ns

*Note*. CVE = Community Violence Exposure; ns = not significant.

As regards the interaction effects, the effect for CVE × Gender was significant, suggesting that the associations of mental health problems with CVE were gender-specific with regard to depressive symptoms and posttraumatic stress, with higher levels in girls. The interaction effect for CVE × Ethnicity was not significant, suggesting that the patterns of mental health problems in relation to the degree of CVE were similar between different ethnic groups. The interaction effect for Gender × Ethnicity was significant, suggesting gender-specific differences in all mental health problems between the ethnic groups (Table 3). Finally, the interaction effect for CVE × Gender × Ethnicity was not significant, although follow-up tests suggested some weakly significant gender-specific associations between CVE and depressive symptoms and posttraumatic stress within ethnic groups.

## Discussion

Until now studies addressing the gender-specific mental health effects of CVE in different ethnic groups have been lacking. This study evaluated the longitudinal associations between CVE and mental health problems from ethnicity and gender perspectives in a large general population sample of inner-city, predominantly ethnic minority adolescents. Significant differences were found in witnessing by ethnicity, with higher rates in African-American and Hispanic-American youth, as compared to Whites, although victimization rates were similar in all three ethnic groups. Girls reported more witnessing of violence, whereas boys reported more direct/violent victimization. Girls in all ethnic groups had significantly higher rates of depressive and posttraumatic stress symptoms, as compared to boys, when experiencing the same degree of CVE as boys. The longitudinal associations between CVE and mental health problems increased similarly over time in line with differences in the degree of CVE severity in all three ethnic groups. The longitudinal associations between CVE and mental health problems were significant even when mental health problems at baseline were controlled for.

In line with previous research, urban adolescents from all three ethnic groups reported a relatively high prevalence of CVE. According to a 2011 U.S. victimization survey, 36.4% of 14 to 17-year-old adolescents witnessed an episode of violence in their community during the past year (Finkelhor et al., 2013). Other studies suggest that at least 50% of urban youth may experience some type of CVE in their neighborhoods (Fowler et al., 2009; Stein et al., 2003). It has been further suggested that the rates of CVE tend to be stable or even increase throughout adolescence, with chronic CVE coming to constitute an everyday reality for urban youth from high-risk neighborhoods, with multiple and repeated traumatic experiences (Gorman-Smith et al., 2004). For example, in one study more than 1 in 10 reported 5 or more exposures in the past year (Finkelhor et al., 2009), while other research has found that up to 75% may be exposed to four or more different violent events during adolescence (Miller et al., 1999). At the same time, in less risky neighborhoods, CVE may occur more randomly, and youth who report CVE may have experienced victimization, but have relatively low levels of witnessing (Zeringue, 2019). This might possibly explain the differences observed in the rates of witnessing found in the present study between White youth and adolescents from other ethnic groups.

While witnessing and victimization in urban populations often co-occur, witnessing is generally more prevalent (Stein et al., 2003), and only a subgroup of youth will experience high levels of victimization (Zimmerman & Posick, 2016), which was true even in the present study. The finding that approximately one-third of all youth reported exposure to at least one episode of victimization in their community during the past year, independent of their ethnic background, is alarming and requires preventive policies and potential treatment measures. Furthermore, the estimates of victimization rates vary broadly even between high risk communities, ranging from 7% (Mrug & Windle, 2009) to 82% (Farrell et al., 2014), most likely because of differences in the types of victimization assessed. Importantly, this suggests that the present study, like many others before it (e.g., Finkelhor et al., 2009), may not have been able to account fully for the multiple victimizations that many youths experience.

The gender differences in witnessing rates in the present study (i.e., the higher rates of witnessing in girls than in boys in all three ethnic groups) may have possibly been related to the definition of victimization used (i.e., at least one reported episode of victimization, regardless of witnessing). Indeed, considering that males are more likely than females to be exposed to different kinds of traumatic events (Finkelhor et al., 2015; Koposov et al., 2021) and that witnessing and victimization tend to be highly correlated in urban samples (Schwab-Stone et al., 2013), it is possible that higher victimization rates in boys “overshadowed” those of witnessing.

The present study demonstrated a step-wise increase in subsequent internalizing mental health problems (posttraumatic stress, depressive symptoms) in relation to an increasing degree of CVE. Indeed, research has indicated that victims of CVE as compared to witnesses are more likely to experience problems with emotion regulation (Schwartz & Proctor, 2000), which in turn may lead to internalizing problems (Dvir et al., 2014). Although some studies have reported that greater levels of CVE in youth may lead to a decrease in depressive symptoms (Fowler et al., 2009), possibly related to desensitization of these youth to the emotional impact of severe and chronic CVE (e.g., Chen et al., 2020; McCart et al., 2007; Sweeney et al., 2011), these findings were not supported by the present study, where we found a clear increase in internalizing problems over time in relation to a greater degree of CVE.

Similarly, the results of the present study are in line with those from several longitudinal studies, which found that even after controlling for initial levels, CVE predicted increases in externalizing behaviors (Hardaway et al., 2016), conduct problems (McCabe et al., 2005; Pearce et al., 2003), aggression (Gorman-Smith & Tolan, 1998), and delinquency and substance use (e.g., Begle et al., 2011; Ford et al, 2010). It has been suggested that witnessing violence and experiencing victimization may lead to increased externalizing behaviors by providing models of antisocial behavior and by affecting individuals’ cognitive processing of social situations (Mrug & Windle, 2009), possibly by increasing hostile attribution bias (Calvete &, Orue, 2011).

Some studies have suggested that witnessing may be more strongly associated with externalizing behaviors than victimization. For instance, witnessing, but not victimization, was found to be associated with antisocial behavior (Bacchini et al., 2015), and predicted subsequent conduct problems and delinquency over time (Mrug & Windle, 2009). In addition, several longitudinal studies have reported a bidirectional relationship between witnessing (but not victimization) and externalizing behaviors (Esposito et al., 2017; Farrell et al., 2014; Mrug & Windle, 2009). However, the above-mentioned proposition was not supported by the results of the current study, where the level of conduct problems similarly increased together with an increasing degree of CVE in all three ethnicities and were not gender-specific. These findings may reflect an important dynamic of CVE, where some youth may report higher levels of exposure because of their own involvement in violence (Gorman-Smith & Tolan, 1998). Indeed, adolescents’ own antisocial behavior may increase the risk of witnessing violence or being victimized (Schwab-Stone et al., 2013), which also, at least to some degree, might help explain the higher rates of internalizing problems seen among antisocial youth (Ruchkin et al., 2002).

Although community violence can affect all ethnic groups, previous research has suggested that both the prevalence of CVE and its mental health outcomes may vary by culture/ethnicity (e.g., Chen, 2010; Schwab-Stone et al., 2013). Indeed, some ethnic groups, such as African-Americans and Hispanic-Americans tend to be disproportionally affected (Crouch et al., 2000; Zimmerman & Messner, 2013), and may even experience more symptoms of distress as a result of their exposure (Berenson et al., 2001; Salzinger et al., 2002). A number of socio-demographic characteristics, such as neighborhood disadvantage (Farrell et al., 2014) and family income (Mrug & Windle, 2009) further impact their levels of CVE. At the same time, other authors have pointed out that while African-American and Hispanic youth may experience greater levels of victimization than White youth, differences in associated internalizing mental health problems may be minimal (Breslau et al., 2004; López et al., 2017). It has been thus postulated that while negative living circumstances are related to a greater risk of exposure, culture is not related to vulnerability when one is exposed and that the magnitude of the relationship between CVE and distress appears to be robust across variations in culture, the amount of exposure, and level of distress (Rosenthal & Wilson, 2006). Similar to other studies (e.g., Rosenthal & Wilson, 2006; Schwab-Stone et al., 2013), the present study suggests that ethnicity is not related to vulnerability when youth are exposed and that the patterns in the relationship between CVE and mental health problems appear to be largely similar across different ethnic groups.

Previous research has shown that in adolescence gender rates of mental health problems tend to diverge, with higher levels of internalizing problems in girls and of externalizing behaviors in boys (Rescorla et al., 2012), and it has even been suggested that this may represent a more general, gender-specific pattern of their reactions to stress (Leadbeater et al., 1999). A number of previous studies have also emphasized the discrepancy, where boys are more likely to be exposed to violence, whereas girls report more internalizing problems related to exposure (e.g., Miliauskas et al., 2022). Yet, some studies have also reported an opposite pattern, with higher levels of externalizing problems in low-income urban girls, as compared to boys (e.g. Carlson & Grant, 2008), speculating that the potential traumatization risk associated with CVE may lead girls to perceive internalizing problems as a less appropriate way of handling stress, and instead opt unconsciously to engage in externalizing behaviors, commonly associated with boys. This notion was not supported by the present study, which found a more typical gender-specific reaction pattern to CVE, with higher levels of depressive and posttraumatic stress symptoms in girls, hence suggesting that this pattern is applicable even to high-risk populations of urban youth, independently of their ethnic background.

Several inferences may be drawn from the present study. First, a greater degree of/proximity to CVE was associated with increased psychopathology, a finding supported by several studies in American inner-city youth (e.g., Buka et al., 2001; Goldner et al., 2015; Fowler et al., 2009; Schwab-Stone et al., 2013), and, as suggested by the present study, this association is generalizable across different ethnicities. Second, independent of their ethnic background, girls tend to react to a similar degree of CVE with greater levels of internalizing problems, compared to boys. This finding of a gender-specific response to trauma has been previously documented in other studies (e.g., Miliauskas et al., 2022; Schwab-Stone et al., 2013), but, as this study suggests, it also seems to be generalizable to different ethnicities. These findings are congruent with the notion of the “universality of trauma responses” in different cultures (e.g., Fletcher, 1997; Saigh, et al., 1999).

Before concluding, several study limitations should be mentioned. First, although the study showed a longitudinal association between CVE and mental health problems even after controlling for the baseline levels of these problems, it was not possible to establish causality. Second, the use of self-reports implies that the data may be subject to recall and reporting biases. Third, the study was based solely on self-reports and other information sources would have increased the validity of the reporting. Finally, while high attrition rates (78% of the original study sample were retained) are typical for urban, ethnic minority, low-income populations, because of their frequent mobility (Lewis & Sinha, 2007), and need to leave the city because of gentrification (Atkinson, 2004), with high school dropout rates reaching up to 50% to 80% in some metropolitan areas (Editorial Projects in Education, 2009), the high level of attrition among the highest-risk youths limits the potential generalizability of our findings. In particular, the students that dropped out of the study reported higher levels of mental health problems than those in the follow-up group and hence, they potentially represent a more vulnerable population whose inclusion in the study might have impacted the results.

## Conclusions

Similar to previous studies, we found that ethnic minority urban adolescents reported high levels of CVE. In addition, this is the first study to suggest that in spite of some differences in CVE between the ethnic groups and by gender, adolescents exposed to a greater degree of violence reported a similar gender-specific pattern of increase in mental health problems over time, regardless of their ethnicity. Appropriate recognition of CVE and associated mental health problems is important for early prevention and intervention strategies, which should be implemented regardless of background, ethnicity, and gender. Although cultural differences may be taken into account when devising these programs, the adverse effects of community violence on adolescents’ mental health may be rather universal. Future longitudinal research on large adolescent general population samples is needed in order to gain a better understanding of CVE rates and their dynamics over time by gender and in different ethnic groups.

## Supplemental Material

sj-docx-1-jiv-10.1177_08862605231158754 – Supplemental material for Longitudinal Associations Between Community Violence Exposure and Mental Health Problems in Inner-City Youth: Ethnicity and Gender Perspectives Click here for additional data file. Supplemental material, sj-docx-1-jiv-10.1177_08862605231158754 for Longitudinal Associations Between Community Violence Exposure and Mental Health Problems in Inner-City Youth: Ethnicity and Gender Perspectives by Vladislav Ruchkin, Johan Isaksson, Andrew Stickley and Mary Schwab-Stone in Journal of Interpersonal Violence
