# Supplementary material for: Longitudinal Associations Between Community Violence Exposure and Mental Health Problems in Inner-City Youth: Ethnicity and Gender Perspectives
Source: J Interpers Violence. 2023 Mar 13;38(13-14):8619–44. doi: 10.1177/08862605231158754 (PMC10326367; doi:10.1177/08862605231158754)

Supplementary Figure 1. Depressive symptoms in year 2 by ethnicity and community violence exposure (CVE) in year 1 in boys and girls


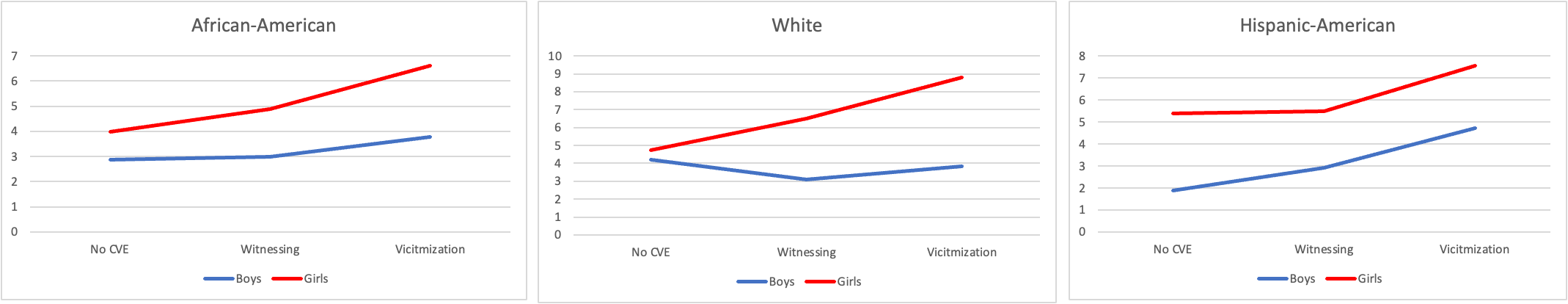


Supplementary Figure 2. Posttraumatic stress in year 2 by ethnicity and community violence exposure (CVE) in year 1 in boys and girls


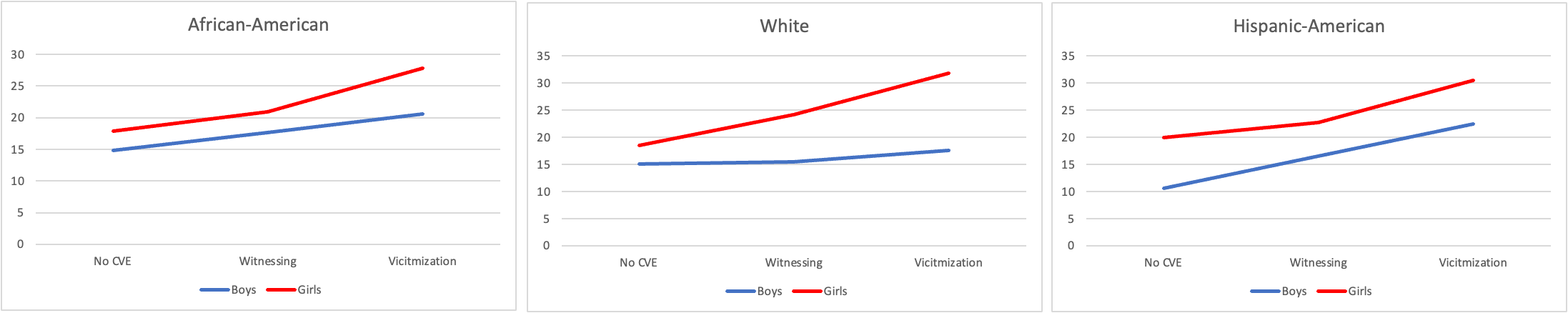


Supplementary Figure 3. Alcohol use in year 2 by ethnicity and community violence exposure (CVE) in year 1 in boys and girls


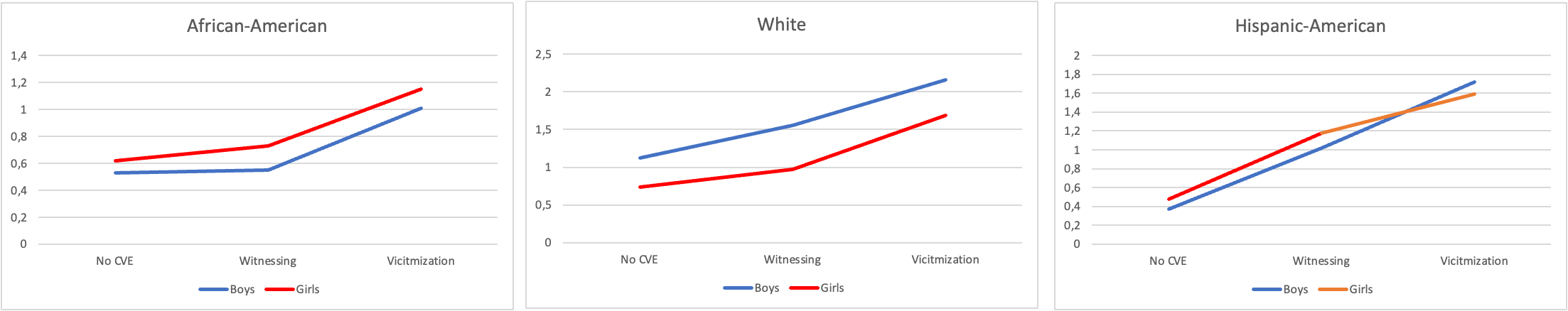


Supplementary Figure 4. Conduct problem in year 2 by ethnicity and community violence exposure (CVE) in year 1 in boys and girls


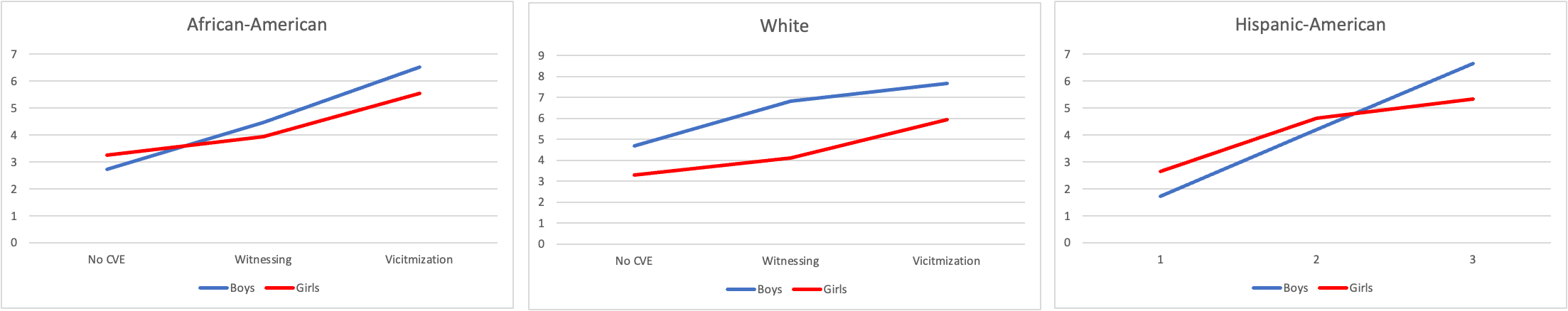

Supplement: sj-docx-1-jiv-10.1177_08862605231158754 – Supplemental material for Longitudinal Associations Between Community Violence Exposure and Mental Health Problems in Inner-City Youth: Ethnicity and Gender Perspectives [file sj-docx-1-jiv-10.1177_08862605231158754.docx]
